# Putative DNA G-quadruplex formation within the promoters of *Plasmodium falciparum var *genes

**DOI:** 10.1186/1471-2164-10-362

**Published:** 2009-08-06

**Authors:** Nicolas Smargiasso, Valérie Gabelica, Christian Damblon, Frédéric Rosu, Edwin De Pauw, Marie-Paule Teulade-Fichou, J Alexandra Rowe, Antoine Claessens

**Affiliations:** 1 Mass Spectrometry Laboratory, GIGA-Research, University of Liege, Liege, Belgium; 2 Structural Biological Chemistry Laboratory, University of Liege, Liege, Belgium; 3 Institut Curie, Section Recherche, CNRS UMR176, Centre Universitaire Paris XI, Bat. 110, 91405 Orsay, France; 4 Centre for Immunity, Infection and Evolution, Institute of Immunology and Infection Research, School of Biological Sciences, University of Edinburgh, Edinburgh, UK

## Abstract

**Background:**

Guanine-rich nucleic acid sequences are capable of folding into an intramolecular four-stranded structure called a G-quadruplex. When found in gene promoter regions, G-quadruplexes can downregulate gene expression, possibly by blocking the transcriptional machinery. Here we have used a genome-wide bioinformatic approach to identify Putative G-Quadruplex Sequences (PQS) in the *Plasmodium falciparum *genome, along with biophysical techniques to examine the physiological stability of *P. falciparum *PQS *in vitro*.

**Results:**

We identified 63 PQS in the non-telomeric regions of the *P. falciparum *clone 3D7. Interestingly, 16 of these PQS occurred in the upstream region of a subset of the *P. falciparum var *genes (group B *var *genes). The *var *gene family encodes PfEMP1, the parasite's major variant antigen and adhesin expressed at the surface of infected erythrocytes, that plays a key role in malaria pathogenesis and immune evasion. The ability of the PQS found in the upstream regions of group B *var *genes (UpsB-Q) to form stable G-quadruplex structures *in vitro *was confirmed using ^1^H NMR, circular dichroism, UV spectroscopy, and thermal denaturation experiments. Moreover, the synthetic compound BOQ1 that shows a higher affinity for DNA forming quadruplex rather than duplex structures was found to bind with high affinity to the UpsB-Q.

**Conclusion:**

This is the first demonstration of non-telomeric PQS in the genome of *P. falciparum *that form stable G-quadruplexes under physiological conditions *in vitro*. These results allow the generation of a novel hypothesis that the G-quadruplex sequences in the upstream regions of *var *genes have the potential to play a role in the transcriptional control of this major virulence-associated multi-gene family.

## Background

*Plasmodium falciparum *is responsible for the majority of malaria cases worldwide and is the cause of an estimated 300–500 million infections and 1–2 million deaths per year [1]. The parasite invades circulating red blood cells and causes them to adhere to microvascular endothelial cells and sequester in blood microvessels, leading to vascular obstruction. The only proteins known to be responsible for this cytoadherence are members of the *P. falciparum *erythrocyte membrane protein one (PfEMP1) family (reviewed in [2]). These highly polymorphic parasite-derived erythrocyte surface proteins are encoded by a repertoire of 50 to 60 *var *genes. Crucially, each parasite expresses only one *var *gene at a time, with transcription sometimes being switched to a different *var *gene in subsequent generations, so allowing antigenic variation and immune evasion [2].

Despite their extreme sequence variability in the coding regions, *var *genes can be divided into 3 major groups (A, B and C) according to the presence of one of three conserved 5' upstream (Ups) sequences (UpsA, UpsB and UpsC) [3]. Their chromosomal position further subdivides them into centromeric (C) or telomeric (T) locations [4]. *Var *gene groups have functional and clinical significance. For example, group B and C *var *genes are known to bind to the endothelial receptor CD36 [2], whereas group A *var *genes have been linked to the most severe clinical forms of malaria [5,6].

The mechanisms regulating *var *gene transcription are not well understood and are currently the subject of intensive investigations. *Var *gene expression is thought to be regulated at the level of transcription initiation [7]. Many mechanisms have been suggested as being involved in the silencing of non-transcribed *var *genes including *var *intron sequences [8] and SPE and CPE motifs located in UpsB and UpsC sequences respectively [9,10]. The histone deacetylase PfSir2 is thought to be required for chromatin silencing in the subtelomeric regions [11], and histone methylation in the 5' Ups region has been shown to regulate transcription of the *var2csa *gene [12]. Finally, a *var*-specific subnuclear expression site has been proposed recently [13]. How these pieces of evidence fit together is still unclear, and other mechanisms may be discovered before the full picture of *var *gene transcriptional control is obtained.

**Figure 1:**
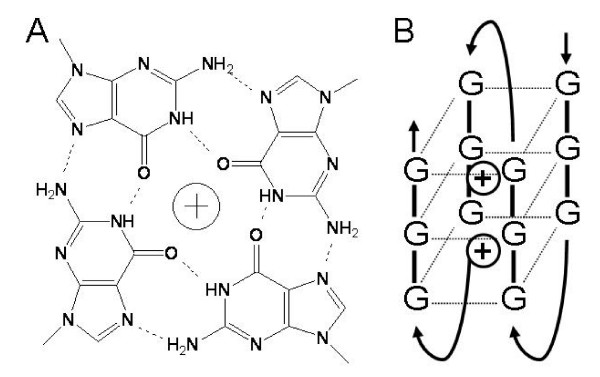
**(A) Chemical structure of a Guanine tetrad**. (B) Schematic representations of a G-quadruplex formed by the folding of a G-rich strand.

DNA usually maintains a double helix structure, however, recent evidence shows that in guanine-rich regions, DNA can adopt a more complex structure called a G-quadruplex [14] (Figure 1). G-quadruplexes are composed by the stacking of guanine tetrads, each one being stabilized by 8 Hoogsteen Hydrogen bonds (Figure 1A). Consequently, sequences containing four groups of three guanines are theoretically able to fold into a G-quadruplex containing three guanine tetrads (Figure 1B). Although there are also a few examples of G-quadruplexes formed from two guanine tetrads [15], these are much less stable and thus less likely to occur *in vivo *[16]. Hence, as in previous genome-wide analyses of potential G-quadruplex-forming sequences [17,18], we chose to investigate here sequences containing at least three tracks of four guanines.

G-quadruplexes are also stabilized by interactions with cations located between the tetrads, at the center of the structure. Potassium and sodium are the most commonly described G-quadruplex stabilizing cations, although ammonium and strontium can also assume this function [19-22]. It was previously reported that there are about 376,000 potential G-quadruplex structures in the human genome [17,18], and about 40% of human genes contain a putative G-quadruplex in their promoter [23]. Initial reports indicate a possible role for G-quadruplex sequences in the regulation of telomere length [24,25] and the transcriptional regulation of several genes such as *c-MYC*, c-kit, or KRAS [23,26-32]. For example, in the case of the *c-MYC *proto-oncogene, a single nucleotide mutation that destabilizes the G-quadruplex structure in the promoter region leads to a three-fold increase in basal transcription levels, suggesting that the G-quadruplex acts as a transcriptional repressor element [27]. Furthermore, a small ligand that binds to and stabilizes the G-quadruplex structure was shown to suppress further *c-MYC *transcriptional activity [27].

Given the increasing evidence for the importance of G-quadruplex sequences in gene regulation, we decided to investigate whether G-quadruplexes could be discovered in the genome of *P. falciparum*, and in particular to determine whether there are any G-quadruplex sequences in the upstream regions of *var *genes that have the potential to play a role in the transcriptional control of this major virulence-associated multi-gene family. In addition, the ability of potential G-quadruplex sequences from *P. falciparum *to form stable G-quadruplex structures under physiological conditions was examined using biophysical techniques.

## Results and discussion

### Identification of putative G-quadruplex forming sequences in the *P. falciparum *genome

The genome of *P. falciparum *clone 3D7 was searched for Putative Quadruplex Sequences (PQS) using QGRS-Mapper [33] on both the positive and negative strands. We set up the QGRS-mapper software to identify all PQS with four repeats of at least three guanines interrupted by loops of a maximum length of 11 nucleotides. As expected, most PQS were found in the telomeres (828 out of 891) due to their repetitive sequence: GGGTT(T/C)A (see Additional file 1). These telomeric G-quadruplexes of *P. falciparum *have recently been described by De Cian *et al *[34]. Here we focused on the non-telomeric PQS because of their potential role in gene transcriptional regulation. We identified 63 non-telomeric PQS (listed in full in Additional file 2). This is an average of one PQS per 380 kb, which is a much lower ratio than that seen in the human genome (1 PQS per ~8 kb) [17]. This was expected due to the extreme AT-richness (80.6%) of the *P. falciparum *genome [35]. 37 of the 63 PQS are in intergenic regions, and of the 26 PQS within genes, 9 are on the coding strand and 17 on the non-coding strand.

### PQS in the upstreamB region of *var *genes

Most importantly, 16 out of the 63 PQS were found in the upstreamB regions of *var *genes, 1612 to 1707 bp upstream of the initiation codon (Table 1 and Additional file 2). These 16 PQS contain only three distinct sequences that thus represent three slightly different putative G-quadruplexes that we named UpsB-Q-1, UpsB-Q-2 and UpsB-Q-3 in order of their frequency (sequences shown in Table 2). As the *var *gene repertoire varies from one clone to another, we also searched for PQS in the upstream regions of *var *genes in *P. falciparum *clone HB3 (available from the Broad Institute website, ). Using the same parameters, we found 11 PQS in the upstream B region of *var *genes in HB3 (Table 1). Interestingly, UpsB-Q-1 was also the most common PQS (7 out of 11) in parasite clone HB3. One PQS was found in HB3 but not in 3D7 (named UpsB-Q-4, Table 1 and 2). These four types of PQS do not exist in any other sequenced organism to date (BLAST analysis, data not shown).

**Table 1:** Predicted G-quadruplex sequences in the upstream regions of Group B *var *genes from *P. falciparum *clones 3D7 and HB3

***P. falciparum *clone 3D7**			***P. falciparum *clone HB3**		
**Gene ID^1^**	***var *group^2^**	**PQS^3^**	**Gene ID^4^**	***var *group^2^**	**PQS^3^**

PF07_0139	BT	Q-1	var14	BT	Q-1
PF08_0142	BT	Q-1	var12	BT	Q-1
PF11_0007	BT	Q-1	var13	BT	Q-1
PF13_0364	BT	Q-1	var20	BT	Q-1
PFA0765c	BT	Q-1	var10	BT	Q-1
PFB1055c	BT	Q-1	var48ψ	BT	Q-1
PFC0005w	BT	Q-1	var49ψ	BT	Q-1
PFC1120c	BT	Q-1	var16	BT	Q-3
PFF1595c	BT	Q-1	var15	BT	Q-3
PFI0005w	BT	Q-1	var8	BT	Q-3
MAL7P1.212	BT	Q-1	var19	BC	Q-4
MAL8P1.220	BT	Q-1			
PF10_0406	BT	Q-2			
PFD1245c	BT	Q-2			
PFL0935c	BC	Q-2			
PFA0005w	BT	Q-3			

^1 ^According to PlasmoDB 5.4 .

^2 ^Var group: B(T/C) = *var *gene with an upstream region B located in telomeric (T) or centromeric (C) region. ψ = pseudogene. [4]

^3 ^PQS: putative quadruplex sequence. The PQS in the upstream B (UpsB) regions were named UpsB-Q-1, UpsB-Q-2 and UpsB-Q-3 in order of their frequency. A 4^th ^PQS found only in HB3 was named UpsB-Q-4.

^4 ^According to Kraemer *et al *[4]

**Table 2:** Sequence of the PQS from the *var *gene upstream B regions of *P. falciparum*

Name	Sequence
UpsB-Q-1	CAGGGTTAAGGGTATAACTTTAGGGGTTAGGGTT
UpsB-Q-2	TAGGGTTAAGGGTATAACGTTAAGGGTTAGGGTT
UpsB-Q-3	CAGGGTTAAGGGTATACATTTAGGGGTTAGGGTT
UpsB-Q-4	CAGGGTTTAGGGTATAACTTTAGGGGTTAGGGTT

### Evidence of G-quadruplex formation by PQS in the upstream B region of *var *genes

**Figure 2:**
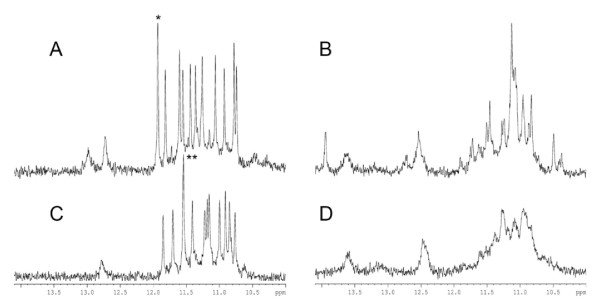
**^1^H NMR spectra of UpsB-Q-1 in potassium (A) and ammonium (C) and of UpsB-Q-2 in potassium (B) and ammonium (D) (cation concentration 150 mM)**. These spectra were acquired at 25°C. *: this peak was resolved in two peaks at 37°C. **: due to its intensity, this peak most likely corresponds to the superposition of two peaks.

In order to confirm the formation of G-quadruplexes by these sequences, two of them (UpsB-Q-1 and 2) were analyzed by ^1^H NMR in the presence of ammonium or potassium ions. Using this technique, it is well established that the presence of imino protons with a chemical shift between 10 and 12 ppm is characteristic of the formation of G-quadruplexes [36-38]. Spectra recorded on the presumably unstructured oligonucleotides in water did not show signals beyond 9 ppm, indicating that imino protons are in fast exchange with bulk water. The spectra recorded after the addition of 150 mM cation are presented in Figure 2 and show clearly the presence of imino protons for the four samples. Most importantly, for UpsB-Q1 in ammonium and potassium, 11 imino peaks are clearly distinguished. In potassium, the peak at 11.93 ppm was resolved in two different peaks at 37°C (data not shown), indicating the formation of a single stable structure containing three quartets. In ammonium, the intensity of the peak at 11.55 ppm could also indicate a superposition of two different peaks. On the UpsB-Q-2 spectra, the imino peaks are less well resolved, indicating some structural polymorphism. Moreover, in both PQS sequences, peaks were observed at higher chemical shift, indicating the possible presence of additional structures like AT-rich hairpins on these G-quadruplexes. These NMR data show that UpsB-Q1 and UpsB-Q-2 do form stable G-quadruplex structures in the presence of physiological concentrations of potassium ions.

**Figure 3:**
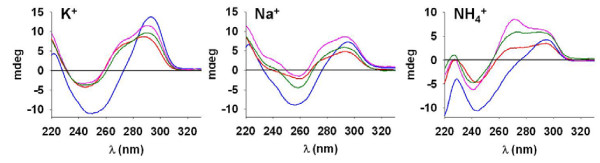
**Circular dichroism spectra of putative G-quadruplex sequences (PQS) from the upstream B regions of *var *genes in 150 mM potassium, sodium and ammonium cation**. UpsB-Q-1 (blue), UpsB-Q-2 (red), UpsB-Q-3 (pink) and UpsB-Q-4 (green). Characteristic signatures of hybrid G-quadruplexes (i.e. containing a mixture of parallel and antiparallel strand orientations) are observed in potassium and ammonium. In sodium, G-quadruplexes are predominantly antiparallel.

The four types of PQS found in the upstream B regions of *var *genes were also examined by circular dichroism (CD), which provides information about the orientation of strands within a G-quadruplex, because the CD signal changes with the syn/anti orientation about the glycosylic bonds. In parallel G-quadruplexes, the CD spectrum typically exhibits a positive peak around 260 nm and a negative peak around 240 nm, whereas in antiparallel G-quadruplexes, the CD spectrum displays a negative peak around 260 nm and a positive peak at 295 nm [39-41]. Results of CD for each UpsB-Q are showed in Figure 3. For UpsB-Q-2, 3 and 4 in potassium and ammonium, a minimum around 243 nm and a maximum around 295 nm were observed, while for UpsB-Q-1 in potassium the minimum was around 250 nm. These kinds of spectra are generally attributed to hybrid conformations (containing a mixture of both parallel and antiparallel strand orientations). In sodium, the four sequences seem to adopt an antiparallel conformation, since a minimum near 260 nm and a maximum at 290 – 295 nm are observed. However, a shoulder was observed in the three cationic conditions at 270–275 nm for UpsB-Q-2, 3 and 4 but not for UpsB-Q-1. The absence of a shoulder in the case of UpsB-Q-1 could therefore indicate a different conformation of this G-quadruplex. Furthermore, the depth of the minimum observed for UpsB-Q-1 was systematically bigger than for the three other sequences. Together with NMR, this result suggests that the single structure adopted by UpsB-Q-1, the most frequent PQS in the UpsB regions, is different (probably closer to an antiparallel form) and that a few base mutations in UpsB-Q-1 (3 bases for UpsB-Q-2, 2 bases for UpsB-Q-3 and a single base for UpsB-Q-4) are sufficient to induce structural polymorphism.

**Figure 4:**
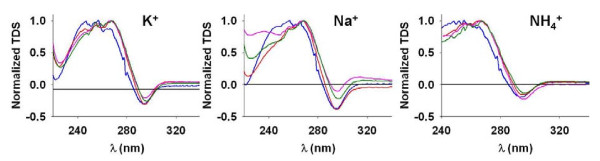
**Thermal denaturation spectra of PQS from the upstream B regions of *var *genes in potassium, sodium and ammonium (cation concentration 150 mM)**. UpsB-Q-1 (green), UpsB-Q-2 (blue), UpsB-Q-3 (red) and UpsB-Q-4 (pink).

Finally, thermal difference spectra (TDS) were recorded [42]. Similarly to CD, this technique differentiates between the various potential structures adopted by DNA. It was shown previously by Mergny *et al *[42] that G-quadruplexes exhibit two positive peaks at 243 and 273 nm and one negative peak at 295 nm, while other DNA structures show different combinations of maxima and minima (see [42] for details of maxima and minima characterizing other DNA structures). The spectra obtained for the PQS from the *var *gene upstream B regions match expectations for G-quadruplex structures (Figure 4). There is a negative peak at 295 nm for all sequences in the three cations. Positive peaks were observed at 246 (only in potassium), 256 (except in ammonium) and 267 nm. These values are in good agreement with previously described results [42] and thus confirm the ability of these sequences to form G-quadruplexes. There is a small wavelength shift for positive peaks, which may be because of the presence of a long loop (10 bases) in the PQS of the *var *gene upstream regions (all the G-quadruplexes previously tested contained no more than four bases in their loops).

The stoichiometries of the G-quadruplexes formed by the UpsB-Q were also examined to determine whether these structures are likely to form intra-molecular bonds (unimolecular structures) or inter-molecular bonds (multimolecular structures) [43]. Mass spectrometry showed only monomeric DNA (Additional file 3), indicating that the UpsB-Q form intra-molecular G quadruplex structures (inter-molecular structures would have been indicated by the presence of multimers by mass spectrometry).

### Stability of G-quadruplexes formed by PQS in the upstreamB region of *var *genes

**Figure 5:**
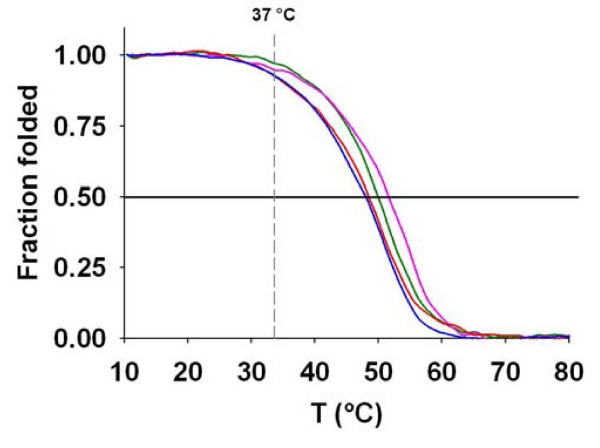
**Thermal denaturation curves (heating) of putative G-quadruplex sequences (PQS) from the upstream B regions of *var *genes in 150 mM potassium cation**. UpsB-Q-1 (blue), UpsB-Q-2 (red), UpsB-Q-3 (pink) and UpsB-Q-4 (green). For the four sequences, *T*_*m*_'s are about 50°C and the proportion of folded G-quadruplexes at 37°C is above 85%.

When the UV absorbance is monitored at 295 nm [44], thermal denaturation experiments allow determination of the *T*_m _of G-quadruplexes, i.e. the temperature at which the half of the signal is lost and thus indicate the relative stability of structures adopted by the oligonucleotides. Because G-quadruplexes with long loops are usually less stable that those with small loops [43,45,46], it was necessary to check if the UpsB-Q are able to form G-quadruplexes that are stable under physiological conditions (37°C and [K^+^] ≈ 150 mM). These results are shown in Figure 4 and the *T*_m_'s are listed in Table 3. In potassium, the four PQS sequences from the *var *gene upstream B regions have a *T*_m _about 50°C, and the transition curves (Figure 5) show that, at 37°C, the proportion of folded G-quadruplexes is higher than 85% for the four sequences, confirming their potential to form G-quadruplexes in living cells. As expected, *T*_m_'s observed in sodium and ammonium are lower than in potassium. This is generally attributed to the weaker stabilization capacity of these two cations, due to their smaller ionic radius [47-49]. The *T*_m _ranking K^+ ^> Na^+ ^> NH_4_^+ ^is also characteristic of antiparallel G-quadruplexes (parallel G-quadruplexes have *T*_m _ranking K^+ ^> NH_4_^+ ^> Na^+^) [43].

**Table 3:** Melting temperature (*T*_*m*_) of G-quadruplex sequences from the upstream B regions of *var *genes and the equilibrium dissociation constant of the PQS with the G-quadruplex ligand BOQ 1 (shown in the K_d _column)

	*T*_*m*_^1^			
		
PQS	K^+^	Na^+^	NH_4_^+^	K_d _(μM)
UpsB-Q-1	47.2 ± 0.6	35.1 ± 1.2	31.6 ± 0.7	2.6 ± 0.5
UpsB-Q-2	49 ± 0.5	36.1 ± 2	27.7 ± 0.5	1.4 ± 0.4
UpsB-Q-3	50 ± 1.2	36.9 ± 1.1	34.2 ± 1.1	1.7 ± 0.7
UpsB-Q-4	49.3 ± 1.4	39.4 ± 0.9	32.3 ± 0.3	2.7 ± 1.1

^1 ^Thermal denaturation experiments were performed at the cation concentration of 150 mM. Thermal denaturation experiments were repeated twice and *T*_*m *_values shown are the mean of four values obtained from four curves (two heatings and two coolings).

### Interactions of UpsB-Q G-quadruplexes with a ligand

In addition to the potential transcriptional repressor activity of G-quadruplex sequences themselves [27], it has been shown previously that G-quadruplex ligands can further suppress transcription of genes containing potential G-quadruplexes in their promoters, by impeding the binding of proteins needed for initiation of transcriptional activity on DNA [50,51]. Moreover, these molecules are also able to interfere with telomere structure and to indirectly induce their shortening [52-55]. These molecules are thus promising weapons in the fight against cancer, since this disease needs both a high expression of oncogenes and stable telomere length to develop and survive [56-62]. With the discovery of G-quadruplex forming sequences in the genome of *P. falciparum*, it can be hypothesized that these ligands may also have the potential to affect parasite gene expression by stabilizing G-quadruplexes located in gene promoter regions.

**Figure 6:**
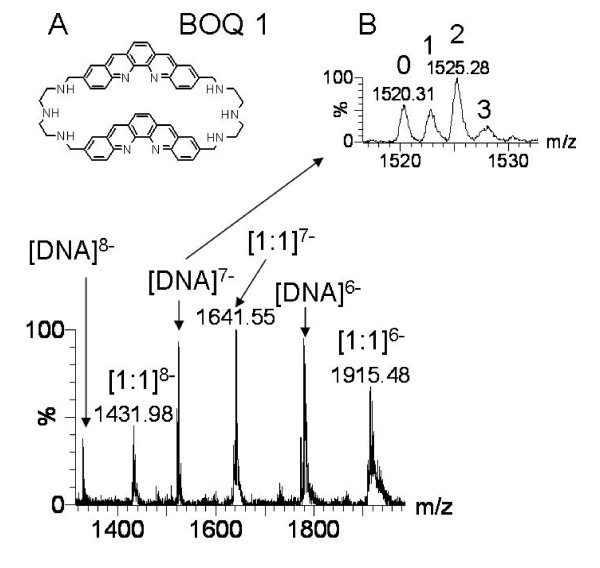
**A: chemical structure of BOQ1**. B: Mass spectrum of UpsB-Q-1 and the G-quadruplex ligand BOQ 1 in equimolar concentrations (10 μM). Only a [1:1] complex was observed. The upper panel shows a magnification on the DNA^7- ^and the number of ammonium adducts (from zero to three). The mass difference between two adjacent ammonium adducts is around 2.43, indicating that the G-quadruplex is intramolecular.

It was thus decided to evaluate the equilibrium dissociation constant of each UpsB-Q with the G-quadruplex ligand BOQ1 (Figure 6A). BOQ1 is a synthetic compound that exhibits a good selectivity for G-quadruplex versus duplex DNA [63,64]. A relatively high association constant with the UpsB-Q sequences would therefore provide additional evidence for the presence of G-quadruplex structures. Binding constants were determined by electrospray mass spectrometry. A typical spectrum of a G-quadruplex-ligand mixture is shown in Figure 6B. The peaks correspond to the free DNA sequence, and to 1:1 complexes (1 ligand per DNA sequence), with charge states of 6-, 7- and 8-. The binding of a second ligand molecule was not observed, revealing that a single binding site is present.

The equilibrium dissociation constants of all UpsB-Q with BOQ 1 were deduced from the relative intensity of peaks of free DNA and complexes, as described previously [65]. For the four sequences, the values are around 2 μM (Table 3). They are lower than those obtained by mass spectrometry for the binding of BOQ1 to telomeric G-quadruplex (5.7 μM) or to model duplex sequences (57 μM) (unpublished data). These results confirm the ability of the PQS in the upstream B regions of the *var *genes to fold in G-quadruplexes, and show that G-quadruplex ligands are likely to bind to these structures within the *P. falciparum *genome, and could therefore be tested for biological activity against the parasite.

### Potential for G-quadruplexes to be involved in gene transcriptional regulation in *P. falciparum*

**Figure 7:**
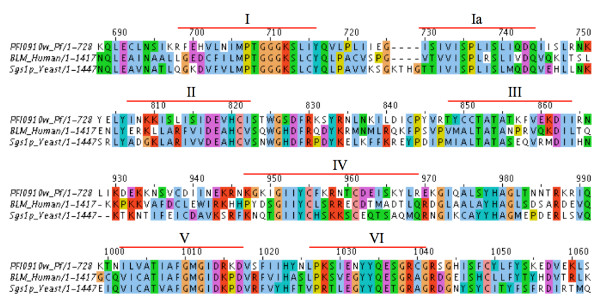
**Alignment of PFI0910w (*P. falciparum*) with two helicases of the RecQ family, BLM (human) and Sgs1p (*Saccharomyces cerevisiae*)**. The seven conserved motifs of the helicase domain are indicated with a red bar.

If the G-quadruplex structures in the *P. falciparum *genome can inhibit gene transcription *in vivo*, one would expect a mechanism to regulate these structures. It has been shown previously *in vitro *that some helicases specifically unwind G-quadruplexes in telomeres [66,67]. Two helicases of the conserved RecQ family, Sgs1p in yeast and BLM in human, show a much higher affinity for G-quadruplex than duplex DNA [68,69]. Moreover, a recent microarray study in a yeast mutant suggests that genes with a PQS in their upstream region tend to be downregulated when Sgs1p is knocked out [70]. The proposed explanation is that, in the absence of the helicase, G-quadruplexes in promoters cannot be unwound and they therefore act as a steric block to the transcriptional machinery [71]. A BLAST search with BLM and Sgs1p in *P. falciparum *brings up PFI0910w, annotated as a putative RecQ helicase on PlasmoDB. A ClustalW alignment with these 3 sequences shows that the 7 domains found in all RecQ helicases are also conserved in PFI0910w (Figure 7). PFI0910w is comparatively shorter and is lacking the RecQ-C-terminal and a HRDC (Helicase-and-RNaseD-like-C-terminal) domain present in BLM or Sgs1p. However, RecQ4, another human helicase part of the RecQ family, is also lacking these 2 domains [72]. Therefore it appears that *P. falciparum *does encode a helicase that may have the capacity to regulate G-quadruplex structures, allowing us to hypothesize that the RecQ helicase (PFI0910w) could be involved in UpsB *var *gene regulation. This suggestion that members of a particular *var *gene group could have a unique regulatory mechanism is not unprecedented, as it has been suggested previously that Group A and E *var *gene transcription, but not groups B and C were influenced by the histone deacetylase Sir2 [11].

## Conclusion

Increasing evidence suggests that G-quadruplexes play a role in gene transcriptional regulation in humans and other organisms. We identified 63 potential G-quadruplex sequences in the non-telomeric regions of the genome of *P. falciparum *clone 3D7. 16 of these PQS occurred in the upstream region of group B *var *genes. The *var *gene-related PQS were shown to form stable G-quadruplex structures *in vitro *under physiological conditions and bind with high affinity to a known G-quadruplex ligand. It is noteworthy that the most prevalent sequence UpsB-Q-1 (dCAGGGTTAAGGGTATAACTTTAGGGGTTAGGGTT) adopts a single structure which is stable in physiological conditions (37°C and 150 mM K^+^). This discovery allows us to generate a new hypothesis concerning *var *gene regulation mechanisms in *P. falciparum*, in which a helicase such as PFI0910w could be involved in G-quadruplex unwinding and thus facilitate RNA polymerase transcriptional activity. The role of G-quadruplexes in *Plasmodium *gene regulation, the structure of these G-quadruplexes, and their use as potential drug targets merits further research.

## Methods

### Bioinformatic analysis

Both strands of each chromosome of the *P. falciparum *3D7 clone (PlasmoDB_5.4 [73]) were analyzed using QGRS-Mapper [33]. The parameters used were: Max length: 33; Min G-group: 3; loop size: 0 to 11. The *P. falciparum *HB3 genome was downloaded from the Broad Institute . Upstream sequences of *var *genes were analyzed using QGRS-Mapper with the same parameters.

### Materials

All oligonucleotides were ordered from Eurogentec (Seraing, Belgium) with Oligold quality. The oligonucleotide sequences used are shown in the Table 2. Oligonucleotides were received lyophilized and stock solutions were prepared in bi-distilled water with 300 μM total strand concentration. For all experiments, the stock solution was heated at 80°C for 5 minutes, diluted using a cold aqueous solution containing either KCl, NaCl or NH_4_OAc to reach the desired DNA concentration in 150 mM cation, and then cooled rapidly on ice. 10 mM lithium cacodylate, pH 7.4 was added in thermal denaturation and circular dichroism experiments. The molecule BOQ1 was synthesized as described previously [74]

### Circular dichroism

Experiments were performed on a Jasco J-810 spectropolarimeter using 1-cm path length cells (Hellma, type No. 114-QS, France). The final concentration of oligonucleotide was 5 μM in a buffer containing 150 mM salt and 10 mM lithium cacodylate, pH 7.4. For each sample, five spectra were recorded from 220 nm to 350 nm with a scan rate of 100 nm/min.

### NMR

NMR samples were prepared by dissolving the oligonucleotides in H_2_O/D_2_O 90/10, lithium cacodylate 10 mM, pH 7.4 to get a oligonucleotide final concentration of 270 μM. Ammonium acetate or potassium chloride were progressively titrated in to a final cation concentration of 150 mM. NMR data were collected at 500 MHz on a Bruker Avance spectrometer (fitted with a TXI triple resonance probe with z-axis gradient). 1D ^1^H spectra were recorded at a temperature of 25°C using a WATERGATE sequence with a water flip-back pulse [75,76].

### Thermal denaturation

Thermal denaturation experiments were carried out on a Uvikon XS spectrophotometer (Secomam), using 1-cm path length quartz cells (Hellma, type No. 115B-QS, France). The final oligonucleotide concentration was 5 μM in 150 mM salt and 10 mM lithium cacodylate, pH = 7.4. Absorbance was monitored as a function of the temperature at 295, 240, 260 nm for the determination of the melting temperature (*T*_m_) [44] and at 405 nm as control wavelength. Gradient was 0.2°C/min between 10 and 90°C. Melting temperatures were determined using the method described by Marky and Breslauer [77]. Before heating and after the cooling, spectra were recorded from 220 to 440 nm, to allow thermal difference spectra (TDS) to be obtained. TDS were obtained by subtracting the low temperature curve from the high temperature curve and normalization, as described previously by Mergny *et al*. [42].

### Electrospray mass spectrometry

All measurements were carried out on a Q-TOF Ultima Global mass spectrometer (Micromass, now Waters, Manchester, U.K.), using the electrospray ionization (ESI) source in negative mode, as described previously [78]. Source conditions were optimized to avoid in-source fragmentation: capillary voltage = -2.2 kV, cone voltage = 50 V, RF, source block temperature = 80°C, and desolvation gas temperature = 100°C. Source backing pressure was set to 3.5 mbar. Oligonucleotide samples were first prepared at 50 μM final concentration in NH_4_OAc 150 mM. Just before injection in the mass spectrometer, they were further diluted to 10 μM in 150 mM NH_4_OAc and 20% methanol. The role of methanol is to increase ion signals.

## Authors' contributions

NS designed research, performed biophysical experiments, analyzed data and wrote the manuscript, AC designed research, performed the bioinformatics analysis, analyzed data and wrote the manuscript. CD performed NMR experiments and wrote the manuscript. FR contributed to biophysical experiments and analyzed data, EDP wrote the manuscript, MPTF contributed new reagent, JAR wrote the manuscript, VG analyzed data and wrote the manuscript. All authors read and approved the final manuscript.

## Supplementary Material

Additional file 1 **Figure s1**. Distribution of telomeric and non-telomeric Putative G-Quadruplex Sequences (PQS) in *Plasmodium falciparum *3D7. Click here for file

Additional file 2 **Figure s2 **Full list of non-telomeric PQS in the Plasmodium falciparum 3D7 genome. Click here for file

Additional file 3 **Supplementary Materials**. Stoichiometry of G quadruplexes formed from the UpsB-Q. Click here for file
