# Supplementary material for: Putative DNA G-quadruplex formation within the promoters of Plasmodium falciparum var genes
Source: BMC Genomics. 2009 Aug 6;10:362. doi: 10.1186/1471-2164-10-362 (PMC2736202; doi:10.1186/1471-2164-10-362)
Supplement: Additional file 1 — Figure s1. Distribution of telomeric and non-telomeric Putative G-Quadruplex Sequences (PQS) in Plasmodium falciparum 3D7. [file 1471-2164-10-362-S1.doc]

**Distribution of telomeric (A) and non-telomeric (B) Putative G-Quadruplex Sequences (PQS) in *Plasmodium falciparum* 3D7.**

**A**

**B**
