# Supplementary material for: Putative DNA G-quadruplex formation within the promoters of Plasmodium falciparum var genes
Source: BMC Genomics. 2009 Aug 6;10:362. doi: 10.1186/1471-2164-10-362 (PMC2736202; doi:10.1186/1471-2164-10-362)
Supplement: Additional file 3 — Supplementary Materials. Stoichiometry of G quadruplexes formed from the UpsB-Q. [file 1471-2164-10-362-S3.doc]

**Stoichiometry of G quadruplexes formed from the UpsB-Q**

It has been shown previously shown that sequences containing four tracks of three guanines are theoretically able to adopt inter-molecular bonds as an alternative to intra-molecular bonds, and could therefore potentially form bimolecular structures [1]. To determine whether the UpsB-Q form intra- or inter-molecular bonds, we used electrospray mass spectrometry to investigate the stoichiometry of the G-quadruplexes formed by UpsB-Q [1]. These experiments were carried out in ammonium acetate using gentle ionization conditions, in order to keep the potential intermolecular structures intact [2-7]. Since ions are characterized by their mass-to-charge ratio in mass spectrometry experiments, it is very simple to determinate the stoichiometry of analytes. Indeed, knowing the molecular mass (M) of the oligonucleotide and given that the charge state (z) of a species could be calculated by measuring the mass-to-charge ratio difference (Δ_m/z_) between two adjacent ammonium adducts by this equation:

z = 17 / Δ_m/z_

the stoichiometry of the structure (n) can be deduced from the mass-to-charge ratio (m/z) using the following equation:

n = [(m/z) × z] / M

We found that only monomeric structures were observed by mass spectrometry (Figure 5A for UpsB-Q-1 and additional figure for UpsB-Q-1, 2, 3 and 4). This therefore indicates that the UpsB-Q sequences form intramolecular G-quadruplexes. This result is not surprising since it was previously demonstrated that the presence of short loops (containing only one or two bases) was required for the formation of multimeric species by sequences containing four tracks of guanines [1], whereas the UpsB-Q have long loops (up to 10 bases).


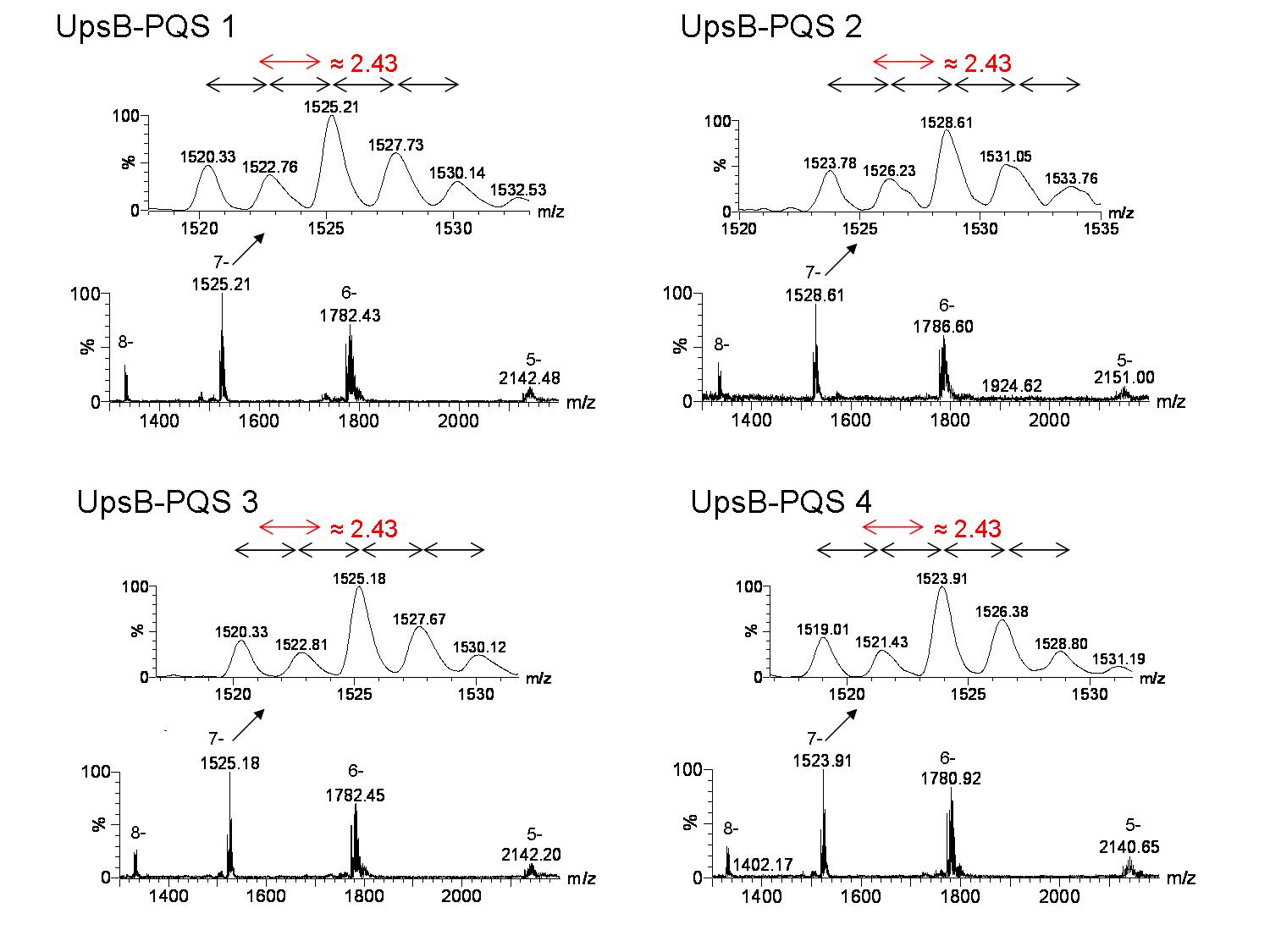


Additional figure: Mass spectra of the four UpsB-Q. The zoom shows that for each sequence, the mass difference between two adjacent ammoniums adducts is 2.43. This difference correspond to a charge state of 7-. Knowing the mass of the oligonucleotides (± 10650 Da), it can be deduce that all the species are composed by only one strand. G-quadruplexes are thus intramolecular.

**References**

1. Smargiasso N, Rosu F, Hsia W, Colson P, Baker ES, Bowers MT, De Pauw E, Gabelica V: **G-quadruplex DNA assemblies: loop length, cation identity, and multimer formation.** *J Am Chem Soc* 2008, **130**:10208-10216.

2. Rosu F, Gabelica V, Houssier C, Colson P, Pauw ED: **Triplex and quadruplex DNA structures studied by electrospray mass spectrometry.** *Rapid Commun Mass Spectrom* 2002, **16**:1729-1736.

3. Baker ES, Bernstein SL, Gabelica V, De Pauw E, Bowers MT: **G-quadruplexes in telomeric repeats are conserved in a solvent-free environment.** *International Journal of Mass Spectrometry* 2006, **253**:225-237.

4. Goodlett DR, Camp DG, 2nd, Hardin CC, Corregan M, Smith RD: **Direct observation of a DNA quadruplex by electrospray ionization mass spectrometry.** *Biol Mass Spectrom* 1993, **22**:181-183.

5. Daniel JM, Friess SD, Rajagopalan S, Wendt S, Zenobi R: **Quantitative determination of noncovalent binding interactions using soft ionization mass spectrometry.** *International Journal of Mass Spectrometry* 2002, **216**:1-27.

6. Rueda M, Luque FJ, Orozco M: **G-quadruplexes can maintain their structure in the gas phase.** *J Am Chem Soc* 2006, **128**:3608-3619.

7. Vairamani M, Gross ML: **G-quadruplex formation of thrombin-binding aptamer detected by electrospray ionization mass spectrometry.** *J Am Chem Soc* 2003, **125**:42-43.
